# A Method to Eliminate the Influence of Frequency-Modulating-Induced Auxiliary Amplitude Modulation on the Calibration of 3 × 3 Coupler Asymmetric Parameters

**DOI:** 10.3390/s20216180

**Published:** 2020-10-30

**Authors:** Yichi Zhang, Jianfei Wang, Mo Chen, Mingyang Wang, Yan Liang, Zhou Meng

**Affiliations:** 1 College of Meteorology and Oceanology, National University of Defense Technology, Changsha 410073, China; gfkdzyc@163.com (Y.Z.); wjfjoy@126.com (J.W.); suiningchenmo@163.com (M.C.); wmypis@126.com (M.W.); liangyan18@nudt.edu.cn (Y.L.); 2 Academy of Artillery and Air Defense, Nanjing 210000, China

**Keywords:** optical fiber sensing system, 3 × 3 demodulation algorithm, calibration of the asymmetric parameters, ellipse fitting, frequency modulation, elimination of the auxiliary amplitude modulation

## Abstract

The 3 × 3 demodulation algorithm has been widely used in retrieving the phase information in the optical fiber sensing system, while the asymmetry of the 3 × 3 coupler can introduce some distortions. In this situation, the parameters of the 3 × 3 coupler can be calibrated by the ellipse fitting method to remove the distortions. Conducting a frequency modulation on the laser, together with an unbalanced Michelson interferometer, makes the ellipse fitting method implemented, which is more appropriate for all-optical sensing. Unexpectedly, the auxiliary amplitude modulation of the laser induced by the frequency modulation is inevitable, leading to the deterioration of the calibration. In this paper, the influence of the auxiliary amplitude modulation on the calibration of the asymmetric parameters is analyzed theoretically and verified experimentally, on the basis of which a convenient but highly efficient method for acquiring the output of the laser and removing related items from the interferometric signals is put forward. The feasibility and robustness of the proposed solution is tested experimentally, and the results show that the mean square errors of the fittings and the variation coefficients of the calibrated parameter sequences are at the scale of $10^{-5}$ and $10^{-4}$, respectively, indicating that the method performs very well.

## 1. Introduction

The 3 × 3 demodulation algorithm is a common methods to demodulate the phase signal of the interferometric optical fiber sensing system [1,2,3,4,5], owning a great deal of advantages such as possessing a high dynamic range and a simple structure compared with the phase generated carrier (PGC) method. However, an effective 3 × 3 demodulation algorithm relies greatly on the strictly symmetric splitting ratio of the 3 × 3 coupler, which can hardly be guaranteed that can lead to the distortion of the retrieved phase signals and the deterioration of the demodulation algorithm [6], limiting the further application of it as a result. Therefore, achieving the 3 × 3 coupler asymmetric parameters before demodulating is crucial in the practical applications, which is called the calibration of the 3 × 3 coupler asymmetric parameters. The ellipse fitting method [7] that fitting the Lissajous figures (LFs) [8] obtained from the interferometric signals with standard ellipses based on the least square method [9], is a common way to obtain the 3 × 3 coupler asymmetric parameters, while the existence of a large time-varying signal is one of the requisite conditions for the formation of the LF.

Two feasible ways can provide the large time-varying signal that the LFs needed: the first one is conducting a large harmonic signal on a piezoelectric ceramic (PZT) embedded in one arm of the balanced Michelson interferometer (the balanced Michelson interferometer is the better choice as the unbalanced one can introduce additional phase noise via the arm difference), which is called the external modulation method (EM) [7]; the second one is conducting a large harmonic frequency modulation on the light source accompanied with an unbalanced Michelson interferometer and it is called the internal modulation method (IM). Despite the fact that the EM method is more convenient intuitively, avoiding the problems brought by the frequency modulation imposed on the light source and the additional phase noise introduced by the unbalanced Michelson interferometer, it is still not appropriate for specific applications (e.g., fiber optic hydrophone) where two arms of the Michelson interferometer act as the probes in the sensing system. In these applications, the electrical signal that the PZT needs impedes the probes from being all-optical, which is particularly vital for an underwater sensor. In this sense, it is a priority to adopt the IM method, with which the all-optical fiber sensing system can be realized. Apparently, the additional phase noise introduced by the indispensable unbalanced interferometer is secondary in the face of such a structural benefit.

Nevertheless, an auxiliary amplitude modulation (AAM) can unexpectedly but inevitably take place in the IM scheme as a frequency modulation (FM) is conducted on the light source [10,11], which leads to the deformations of the desired elliptical-shape LPs and makes the calibration fail to perform well. The phenomenon is more noteworthy for the semiconductor laser (SL) in which the FM is realized by conducting the radio-frequency current on it directly. In previous reports, studies concerning the AAM are mainly dedicated to the influence of it on the PGC demodulation algorithm and the suppression methods [12,13]. He et al. [12] put forward a novel ameliorated PGC algorithm based on arctangent function and differential-self-multiplying which can keep stable operations with light intensity disturbance, which is relatively complicated. Shi et al. [13] measured the depth and phase delay of the AAM, observed the stability of the AAM parameters and removed it straightforwardly from the PGC interference signal, which merely took the first harmonic into consideration. Sweeney et at. [14] recently proposed a method of amplitude compensation to overcome the effects of radiation-induced attenuation in the application fields of versatile test reactor, in which the 3 × 3-coupler-based Michelson interferometer acts as the interrogation interferometer rather than a probe. However, their method fails to take the deviation of the phase difference into consideration, which is the drawback compared with the ellipse fitting method. Generally, the study on the effect of AAM on calibrating the 3 × 3 coupler asymmetric parameters with the ellipse fitting method and its suppression has never been reported to the best knowledge.

In the paper, the influence of the abovementioned AAM on the calibration of the 3 × 3 coupler parameters is studied theoretically and experimentally, while a method that detects the output of the light source with a 1:99 coupler and removes it in real time from all the interferometric signals to eliminate the negative effect of the AAM is put forward. Additionally, the ratio between the acquired AAM and the actual AAM imposed on the interference signals, which can hardly be confirmed, is demonstrated to have no influence on the 3 × 3 demodulation algorithm. Ultimately, the proposed treatment is verified experimentally, the results of which show that such a solution possesses a great feasibility and robustness. The works in the paper provide a simple but highly efficient approach to improving the performance of the IM-based calibration of the 3 × 3 coupler parameters.

## 2. Theory and Simulation

According to the coupling-mode theory, the three interferometric signals of the 3 × 3-coupler-based Michelson interferometer as the light source is conducted an FM can be expressed, respectively, as follows [15]:

(1) $$\{\begin{array}{c} \mu_{1}=A_{1}+B_{1}\cos\left[C\cos\left(\omega_{m}t+\phi_{0}\right)+\varphi \left(t\right)+\varphi_{0}\right]\\ \mu_{2}=A_{2}+B_{2}\cos\left[C\cos\left(\omega_{m}t+\phi_{0}\right)+\varphi \left(t\right)+\varphi_{0}+\phi_{12}\right]\\ \mu_{3}=A_{3}+B_{3}\cos\left[C\cos\left(\omega_{m}t+\phi_{0}\right)+\varphi \left(t\right)+\varphi_{0}-\phi_{13}\right] \end{array},$$

where $\mu_{1}$, $\mu_{2}$, $\mu_{3}$ are the output voltages of the three photoelectric detectors (PD), $A_{1},\;A_{2},\;A_{3}$ are the zero-frequency components of the three-channel interferometric signals, *B*_1_, $B_{2}$, $B_{3}$ are the amplitudes of the alternating components (AC) of the interferometric signals. The FM modulation depth item C is equal to Δkn2L=4πnLΔν/c in the IM scheme, in which n is the refractive index of the fiber, L is the arm difference of the unbalanced Michelson interferometer, Δν is the maximal modulation amplitude of the optical frequency and c is the speed of light. The item $\omega_{m}$ is the modulation frequency of the FM, while $\phi_{0}$ is the initial phase of the FM. The item φ(t) is the phase information imposed on the arms of the Michelson interferometer, which is the one that 3 × 3 demodulation algorithm extracts from the interferometric signals. $\varphi_{0}$ is the initial phase, $\phi_{12}$ and $\phi_{13}$ are the fixed phase differences resulting from the characteristic of the 3 × 3 coupler. In the ideal case, $A_{1}=A_{2}=A_{3}$, $B_{1}=B_{2}=B_{3}$, and $\phi_{12}=\phi_{13}=120^{{}^{\circ}}$ and the phase information φ(t) can be conveniently retrieved by the symmetric demodulation algorithm based on 3 × 3 coupler presented in [16].

However, due to the practical asymmetry of the 3 × 3 coupler splitting ratio and the diverse conversion efficiencies of the PDs, the relationships of the parameters turn to be $A_{1}\neq A_{2}\neq A_{3}$ and $B_{1}\neq B_{2}\neq B_{3}$, while $\phi_{12}$ and $\phi_{13}$ fail to be constants and deviate from $120^{{}^{\circ}}$ with the change of the environment as well as the polarization state of the light, which can bring a degree of distortion into the retrieved phase signal [6]. Hence, figuring out the abovementioned time-varying parameters before the demodulation is an indispensable procedure, which is called the calibration of the 3 × 3 coupler asymmetric parameters. It is observed from Equation (1) that any two channels can constitute an elliptic-shaped LF as they share the same modulation and possess a relatively fixed phase difference. Thus, the process of the calibration can be described as follows [7]: first, plot the scatter diagram with the datapoints of any two interferometric signals; second, fit the scatter diagram with a standard ellipse; third, obtain the asymmetric parameters $A_{1}$, $A_{2}$, $A_{3}$, $B_{1}$, $B_{2}$, $B_{3}$ as well as $\phi_{12}$ and $\phi_{13}$ from the coefficients of the fitted elliptic equation.

Nevertheless, the influence of the AAM is neglected in the above analysis, which is so serious that it can damage the normal calibration of the parameters in the way described as follows: when the laser is imposed on an FM with a frequency $\omega_{m}$, the unexpected AAM exerts the same degree of effects on the six parameters $A_{1}$, $A_{2}$, $A_{3}$ and $B_{1}$, $B_{2}$, $B_{3}$, as they are all affected directly by the output power of the light source, meaning that they fluctuate with the amplitude-modulated output of the laser. In this circumstance, the three interferometric signals shown in Equation (1) should be modified as follows:

(2) $$\left\{ \begin{array}{c} \mu_{1}=\left(1+\overset{\infty }{\underset{p=1}{\sum }}M_{p}\cos p\omega_{m}t\right)\left\{A_{1}+B_{1}\cos\left[C\cos\left(\omega_{m}t+\phi_{0}\right)+\varphi \left(t\right)+\varphi_{0}\right]\right\}\\ \mu_{2}=\left(1+\overset{\infty }{\underset{p=1}{\sum }}M_{p}\cos p\omega_{m}t\right)\left\{A_{2}+B_{2}\cos\left[C\cos\left(\omega_{m}t+\phi_{0}\right)+\varphi \left(t\right)+\varphi_{0}+\phi_{12}\right]\right\}\\ \mu_{3}=\left(1+\overset{\infty }{\underset{p=1}{\sum }}M_{p}\cos p\omega_{m}t\right)\left\{A_{3}+B_{3}\cos\left[C\cos\left(\omega_{m}t+\phi_{0}\right)+\varphi \left(t\right)+\varphi_{0}-\phi_{13}\right]\right\} \end{array},\right.$$

where $M_{p}$ is the modulation depth of the pth-order harmonic, and the newly multiplied item represents the influence of AAM, which consists of fundamental frequency component and its high-order harmonics component. It should be pointed out here that the AAM item in Equation (2) is normalized reasonably since the initial intensity of the laser is included in the six asymmetric parameters. It is implied in Equation (2) that the newly generated asymmetric parameters can be expressed as $A_{1}^{\prime }=i_{AAM}A_{1}$, $B_{1}^{\prime }=i_{AAM}B_{1}$, $A_{2}^{\prime }=i_{AAM}A_{2}$, $B_{2}^{\prime }=i_{AAM}B_{2}$, $A_{3}^{\prime }=i_{AAM}A_{3}$, $B_{3}^{\prime }=i_{AAM}B_{3}$, in which

(3) $$\begin{array}{c} i_{AAM}=1+\overset{\infty }{\underset{p=1}{\sum }}M_{p}\cos p\omega_{m}t. \end{array}$$

Considering that the six asymmetric parameters mainly have effects on the size of the elliptic-shaped LFs and they are all changed with the rhythm totally identical to that of $i_{AAM}$, the size of the ellipses in the LFs can expand and shrink sinusoidally with the slant angle of the long axis unchanged.

**Figure 1:**
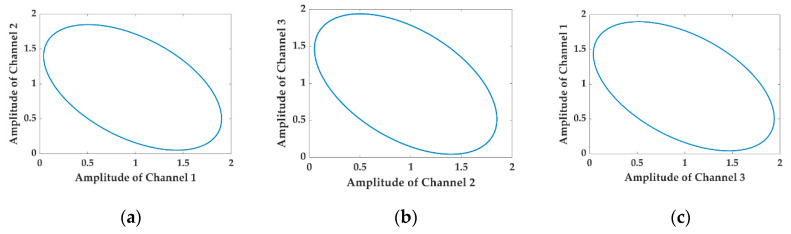
Numerical results obtained from Equation (1), in which the auxiliary amplitude modulation (AAM) of the light source is neglected: standard ellipses. (**a**) The Lissajous figure (LF) drawn with Channel 1 and Channel 2; (**b**) the LF drawn with Channel 2 and Channel 3; (**c**) the LF drawn with Channel 3 and Channel 1.

**Figure 2:**
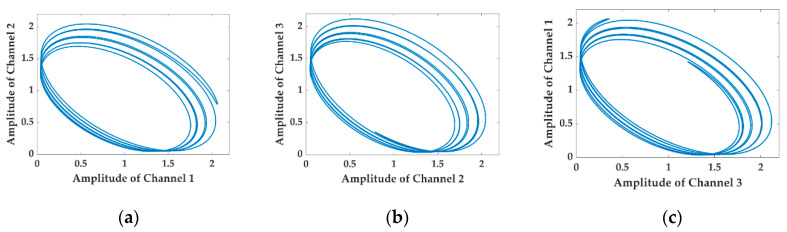
Numerical results obtained from Equation (2), in which the AAM of the light source is taken into consideration: fail to maintain the elliptic line anymore. (**a**) The LF drawn with Channel 1 and Channel 2; (**b**) the LF drawn with Channel 2 and Channel 3; (**c**) the LF drawn with Channel 3 and Channel 1.

To simplify the analysis, the harmonics higher than 2nd-order are neglected in the numerical simulation, and the coefficients in Equations (1) and (2) are set as follows: $A_{1}=0.97$, $A_{2}=$ 0.95, $A_{3}=0.99$, $B_{1}=0.93$, $B_{2}=0.9$, $B_{3}=0.95$, $M_{1}=0.1$, $M_{2}=0.01$, $\omega_{m}=2\pi \times 500\;\mathrm{Hz}$, C=12, $\phi_{0}=18^{{}^{\circ}}$, $\varphi_{0}=18^{{}^{\circ}}$, $\phi_{12}=\phi_{13}=120^{{}^{\circ}}$, φ(t)≡0. The numerical results of the LFs drawn with the interferometric signals in Equations (1) and (2) are shown in Figure 1 and Figure 2, respectively.

It is observed by comparing Figure 1 with Figure 2 that the LFs in Figure 2 fail to remain as the original standard ellipses shown in Figure 1 as the AAM item is conducted on the three interferometric signals. Instead, they deform to be disordered shapes in which a series of ellipses with various coefficients appear, precisely resulting from the conducting of the AAM item. In such a terrible case, the fitted elliptic equation cannot represent the internal regularity of the datapoints anymore, and the asymmetric parameters of the 3 × 3 coupler achieved from the elliptic coefficients are far from credibility.

Therefore, solving the encountered AAM problem is the prerequisite for the calibration of the 3 × 3 asymmetric parameters. Taking the source and the action mechanism of the AAM into consideration, one feasible and highly efficient solution is to detect the output of the laser and get rid of the influence of the AAM from the three interferometric signals in Equation (2) directly. The output of the laser contains the AAM item as well, which can be expressed as:

(4) $$\begin{array}{c} \mu_{output}=\xi \left(1+\overset{\infty }{\underset{p=1}{\sum }}M_{p}\cos p\omega_{m}t\right), \end{array}$$

where the coefficient ξ, which is hardly to determine precisely, is the product of the photoelectric conversion efficiency of the PD, the splitting ratio of the coupler through which the output of the laser is split, and other attenuation on the transmitting pathway to the input port of the 3 × 3 coupler. Ultimately, after being divided by $\mu_{output}$ in Equation (4), Equation (2) turns to be:

(5) $$\left\{ \begin{array}{c} \mu_{1}^{\prime }=A_{1}/\xi +B_{1}/\xi \cos[C\;\cos(\omega_{m}t+\phi_{0})+\varphi (t)+\varphi_{0}]\\ \mu_{2}^{\prime }=A_{2}/\xi +B_{2}/\xi \cos[C\;\cos(\omega_{m}t+\phi_{0})+\varphi (t)+\varphi_{0}+\varphi_{12}]\\ \mu_{3}^{\prime }=A_{3}/\xi +B_{3}/\xi \cos[C\;\cos(\omega_{m}t+\phi_{0})+\varphi (t)+\varphi_{0}-\varphi_{13}] \end{array}.\right.$$

It is observed in Equation (5) that the original six parameters in Equations (1) and (2) are affected identically by the fixed coefficient ξ after being divided by the acquired $\mu_{output}$, which makes the precious LFs shrink (or expand) to new similar ellipses time independently, that is, the newly formed LFs turn back into ellipses. Correspondingly, the results achieved from the calibration are transformed into $A_{1}^{\prime }=A_{1}/\xi$, $B_{1}^{\prime }=B_{1}/\xi$, $A_{2}^{\prime }=A_{2}/\xi$, $B_{2}^{\prime }=B_{2}/\xi$, $A_{3}^{\prime }=A_{3}/\xi$, $B_{3}^{\prime }=B_{3}/\xi$, $\phi_{12}$ and $\phi_{13}$, respectively.

In the end, it is worth noting that the newly introduced coefficient ξ has no influence on the subsequent demodulation algorithm described in [7], in which the coefficient ξ is blended into the measured parameters $A_{1}^{\prime }$, $B_{1}^{\prime }$, $A_{2}^{\prime }$, $B_{2}^{\prime }$, $A_{3}^{\prime }$, $B_{3}^{\prime }$ and removed from the interferometric signals integrally, as getting rid of the direct component (DC) items and AC items is originally an indispensable step of the demodulation algorithm.

## 3. Experimental Setup

**Figure 3:**
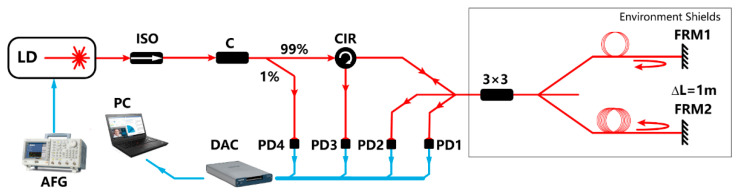
The experimental setup for researching the influence of the AAM and its suppression.

In order to research the influence of the AAM experimentally and validate the solution proposed, an experimental setup is constructed as is illustrated in Figure 3. The light source is a diode laser (LD, Orion^™^ series, RIO) centered at 1550 nm, providing an output power of 10 mW, followed by an optical fiber isolator (ISO) to avoid the influence of the echo. A sinusoidal modulation signal is generated by an arbitrary waveform generator (AWG) which is conducted on the light source to implement the FM. The label C represents a 1:99 2 × 2 coupler—the output of the laser is acquired by the 1% beam and achieved by PD4, which possesses a low circuit noise. The role of the circulator (CIR) is to make the third interferometric signal able to be detected by PD3 without hindering the transmission of the input light. The two arms at the opposite side of the 3 × 3 coupler, which own a difference in the length, constitute an unbalanced Michelson interferometer. The Faraday rotator mirror (FRM) eliminates the influence of the polarization fade by making the polarization change of the reflected light precisely counteract that of the incident light [17]. The unbalanced Michelson interferometer together with the frequency modulated laser contributes to the realization of the IM method. Besides Channel 3, the other two channels of the interferometric signals are detected by another two low noise photodetectors which are named by PD1 and PD2, respectively, and processed by a personal computer (PC) after being acquired from a digital acquisition card (DAC). The 3 × 3 coupler together with the arms of the interferometer is placed in an environment shield to cut off the external disturbance. To be noted, the length difference between the two arms of the Michelson interferometer in the experiment is 1 m, and the corresponding optical path difference (OPD) is 2.912 m considering that the light experiences a round-trip transmission in each arm. Considering that the laser output is acquired via the 1% branch of the 1:99 coupler, the gain of the PD4 is set to be 10 times higher than other three in order to prevent the background circuit noise level of the PD4 from being comparable to electrical signal amplitude converted from the acquired light, which can lead to the thickening of the LFs due to the noise-induced random fluctuation.

## 4. Experimental Results and Discussions

**Figure 4:**
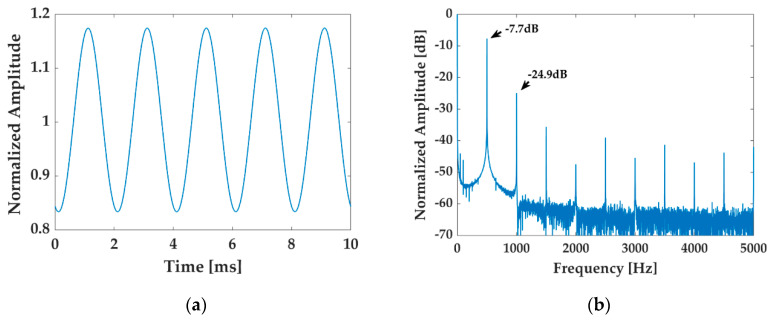
Normalized results of the light source output. (**a**) The time-domain waveform; (**b**) the logarithmic frequency spectrum.

The modulation frequency of the FM being set as $f_{m}=500\;\mathrm{Hz}$ and the depth as C=12, the three interferometric signals from the 3 × 3 coupler are acquired on basis of the experimental setup, so is the output of the laser. The normalized results of the laser output are shown in Figure 4, in which all the data are divided by the zero-frequency value to make the distinction between the DC and the AC more intuitive. The Figure 4a shows the normalized time-domain wave diagram of the frequency-modulated laser output, indicating that the amplitude of the output is modulated by the AAM, while the frequency component of $f_{m}=500\;\mathrm{Hz}$ and its higher harmonics are observed in the logarithmic frequency spectrum of the laser output, as is shown in Figure 4b. Additional attention should be paid on the amplitudes at the frequency $f_{m}$ and $2f_{m}$, which are only 7.7 dB and 24.9 dB lower than the DC component, respectively, revealing that the AAM is so drastic that can have a non-negligible influence on the calibration.

**Figure 5:**
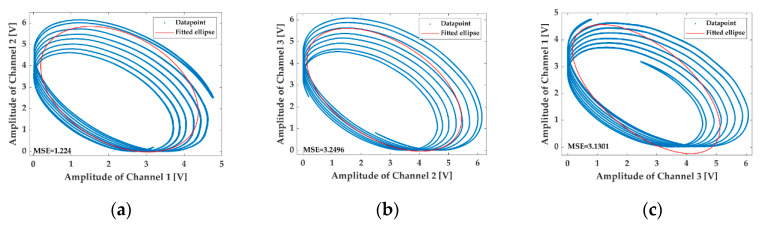
The LFs drawn by the experimentally acquired interferometric signals of the 3 × 3 coupler. (**a**) The LF drawn with Channel 1 and Channel 2; (**b**) the LF drawn with Channel 2 and Channel 3; (**c**) the LF drawn with Channel 3 and Channel 1.

The practical LFs obtained from the experimental data, which are illustrated in Figure 5, also demonstrate that the effect of the AAM on the calibration of the 3 × 3 coupler parameters cannot be neglected as the LFs are made up of a series of ellipse with various sizes instead of standard stable ellipses, being nearly consistent with the results of the numerical simulation shown in Figure 2. The fitting errors can be measured by the mean square errors (MSEs), which can be expressed by [18]

(6) $$\begin{array}{c} MSE=\frac{1}{m}\overset{m}{\underset{i=1}{\sum }}{\left(y_{i}-{\hat{y}}_{i}\right)}^{2}, \end{array}$$

where $y_{i}$ is the y-coordinate of the measured datapoint $x_{i}\left(x_{i},\;y_{i}\right)$, ${\hat{y}}_{i}$ is the calculated y-coordinate by substituting the x-coordinate $x_{i}$ into the fitted standard elliptic equation, and m is the number of the datapoint. It is displayed in the Figure 5 that the MSEs of the fitting are 1.2240, 3.2496, 3.1301 respectively in the AAM-influenced situation. Apparently, fitting such unexpected patterns of the LFs with standard ellipses can introduce considerable errors into the calibration of the 3 × 3 coupler asymmetric parameters.

**Figure 6:**
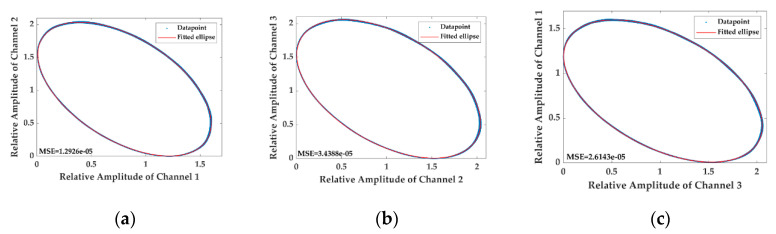
The LPs drawn with the processed interferometric signals and the fitted ellipses. (**a**) The LF plotted with the relative amplitude of Channel 1 and Channel 2 as well as the fitted ellipse; (**b**) the LF plotted with the relative amplitude of Channel 2 and Channel 3 as well as the fitted ellipse; (**c**) the LF plotted with the relative amplitude of Channel 3 and Channel 1 as well as the fitted ellipse.

The feasibility of the proposed solution in eliminating the influence of the AAM on the calibration of the 3 × 3 coupler asymmetric parameters is verified experimentally. In the experiment, the three channels of the interferometric signals acquired from PD1, PD2 and PD3 are all divided by the output of the light source achieved from PD4. As a result, another three interferometric signals are generated, which possess the form of the signals in Equation (5) without any correlations with the AAM according to the theory section. Three pieces of LFs are drawn with the processed interferometric signals to validate the practical performance of the handling method, which are displayed in Figure 6. It is manifested that shapes of the LFs are close to ellipses after the interferometric signals are divided by the acquired output of the laser, which can be fitted well with the standard ellipses as the MSEs of the fitting results are about $1.29\times 10^{-5}$, $3.44\times 10^{-5}$ and $2.61\times 10^{-5}$, respectively, which are all almost five orders of magnitude lower than the mean value of the processed interferometric signals ($\sim 10^{0}$), indicating that the influence of the AAM is nearly wiped out and the precision of the calibration can reach a high level.

**Figure 7:**
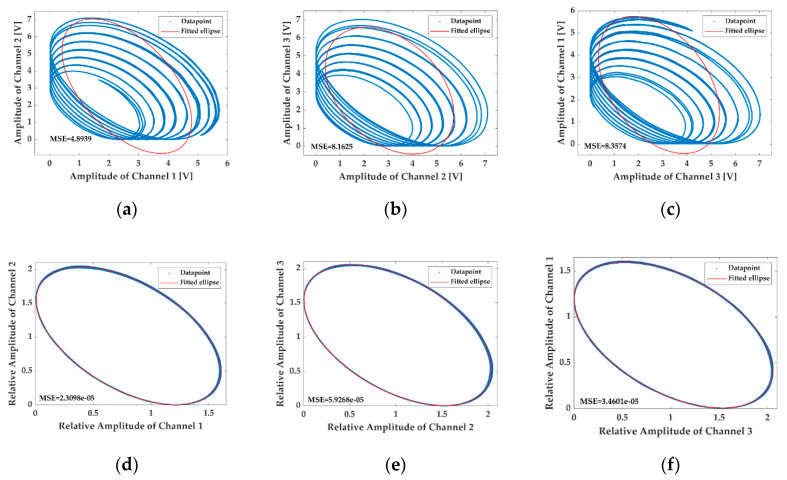
The LFs drawn with the interferometric signals on the frequency modulation (FM) condition of $f_{m}=500\;\mathrm{Hz}$ and C=24 before (**a**–**c**) and after (**d**–**f**) removing the influence of AAM by the proposed method.

**Figure 8:**
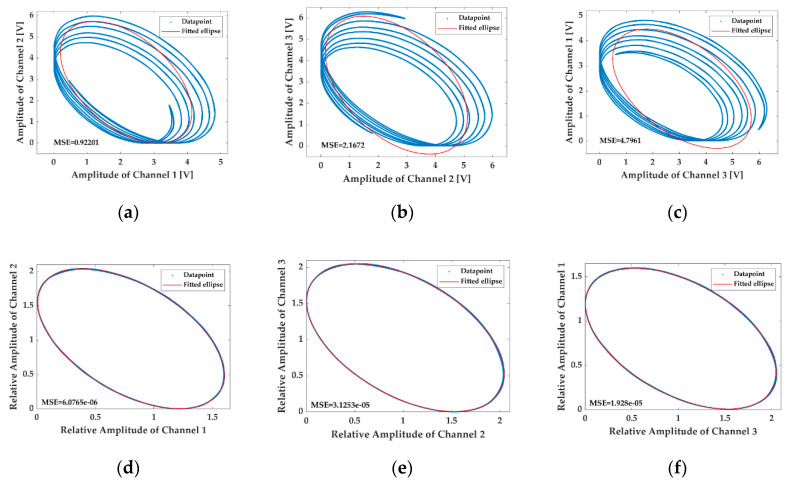
The LFs drawn with the interferometric signals on the FM condition of $f_{m}=1000\;\mathrm{Hz}$ and C=12 before (**a**–**c**) and after (**d**–**f**) removing the influence of AAM by the proposed method.

The modulation conditions are then changed to be $f_{m}=500\;\mathrm{Hz}$ and C=24, and the LFs obtained from the interferometric signals before and after removing the influence of AAM by the proposed method are illustrated in Figure 7, while Figure 8 shows the results under the circumstances of $f_{m}=1000\;\mathrm{Hz}$ and C=12. Intuitively, the negative influence of the AAM on the calibration increases with the modulation depth C, but can hardly be changed with the modulation frequency $f_{m}$. Additionally, it is observed in Figure 7b,c that the influence of the AAM is so drastic in the Figure 7 situation that the MSEs of the fitting are even higher than the maximal magnitudes of the interferometric signals. However, no matter how serious the effects of the AAM on the ellipse fittings are, the proposed solution can always make the awful shapes of the LFs back to nearly standard ellipses, and the MSEs of the fitting are all at $10^{-5}$ scale or even lower, indicating that the proposed handling method possesses an excellent feasibility and robustness.

**Figure 9:**
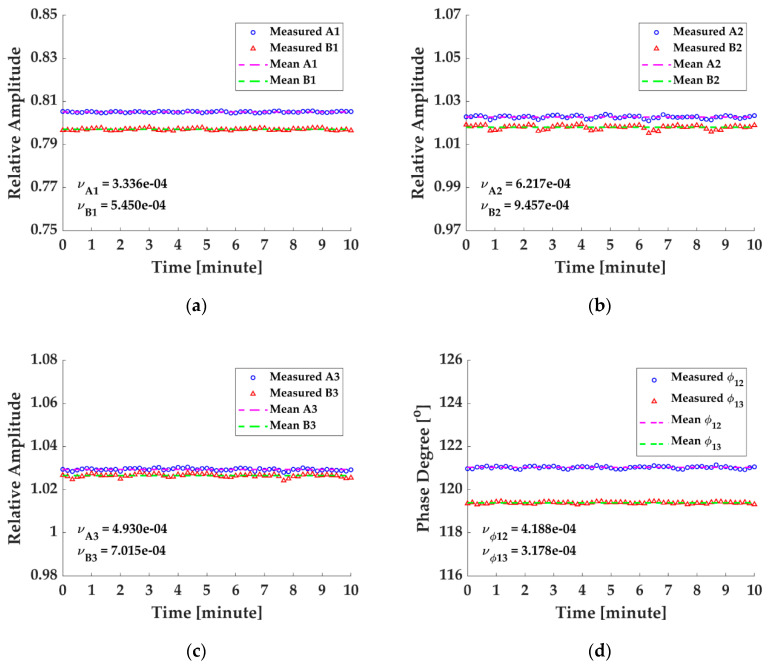
Stability of the calibrated parameters in the 10-min test: (**a**) the relative amplitude of the calibrated parameters $A_{1}$, $B_{1}$ and their mean values; (**b**) the relative amplitude of the calibrated parameters $A_{2}$, $B_{2}$ and their mean values; (**c**) the relative amplitude of the calibrated parameters $A_{3}$, $B_{3}$ and their mean values; (**d**) the magnitudes of the calibrated phase degree parameters and their mean values.

In addition, the stability of the ameliorated calibration method with the influence of the AAM being suppressed is monitored on the condition of $f_{m}=1000\;\mathrm{Hz}$, C=12 and the temperature being kept at 26 °C. The asymmetric parameters are calibrated with the ameliorated method every 10 s and the test lasts 10 min totally. The monitoring results are illustrated in Figure 9, in which it is observed that the parameters are reasonably stable intuitively, while the variation coefficients (the ratio of the standard derivation to the mean value) [19] of the acquired parameter sequences are also figured out to quantitatively analyze the stability of the calibration method, which are all at $10^{-4}$ scale ($v_{A1}=3.336\times 10^{-4}$, $v_{B1}=5.450\times 10^{-4}$, $v_{A2}=6.217\times 10^{-4}$, $v_{B2}=9.457\times 10^{-4}$, $v_{A3}=4.930\times 10^{-4}$, $v_{B3}=7.015\times 10^{-4}$, $v_{\phi_{12}}=4.188\times 10^{-4}$, and $v_{\phi_{12}}=3.178\times 10^{-4})$, proving quantificationally that the improved calibration method is practically stable and feasible. Besides the above findings, it also should be mentioned that the minor fluctuations of the calibrated parameters can ascribe not only to the ellipse fitting error, but also to the tiny change of the polarization state [20], as all the optical fiber component used in the experiment are not polarization maintaining, and the influence of the polarization state on the 3 × 3 coupler asymmetric parameters needs deeper studies.

Furthermore, the simple but highly efficient solution to the AAM influenced interferometric signals proposed in the paper also performs very well in inhibiting the effects of other intensity noise originated from the light source on the calibration of the 3 × 3 coupler asymmetric parameters, such as the intensity noise results from relaxation oscillation and the power-line interference.

In summary, the proposed solution of making the interferometric signals divided directly by the acquired time-domain waveform of the laser output works well in eliminating the influence of the AAM on the calibration of the 3 × 3 coupler asymmetric parameters, providing a simple, highly efficient, robust method to enhance the precision of the calibration and paving the way for avoiding the unexpected distortion in the 3 × 3 demodulation procedure. The method put forward can be directly and universally applied before the practical phase demodulation process in the phase-modulation-type optical fiber sensing system.

## 5. Conclusions

In the paper, the influence of the AAM on the calibration of the 3 × 3 coupler asymmetric parameters adopting the IM scheme is analyzed theoretically, and the numerical results show that the LFs drawn with the AAM-influenced interferometric signals of the 3 × 3-coupler-based Michelson interferometer cannot remain to be elliptic shaped, but reform to be abnormal forms overlapped with a series of ellipses with various coefficients, which cannot reveal the intrinsic patterns of the datapoint, and the ellipse fitting method thereby loses its efficacy. A method for obtaining the laser output from the input path and removing it from the AAM-influenced interferometric signals by a simple division is put forward based on the theoretical analysis, while a constant coefficient is introduced into all the interferometric signals, which is theoretically proved to exert no influence on the subsequent demodulation processing in the end. Relevant experiments are carried out to validate the influence of the AAM on the calibration and verify the practical feasibility of the proposed handling method. The AAM is ascertained as nonnegligible experimentally, as the amplitudes of the fundamental frequency and 2nd harmonic component are only 7.7 dB and 25 dB lower than the DC component respectively. Based on the validation, it is demonstrated that the LFs drawn with the practical interferometric signals generated from the IM mode can deviate considerably from ellipses—the patterns of which show high similarity to those achieved from the numerical simulation. Therefore, the ellipse fitting method is inappropriate under such a circumstance. The handling method raised in the theoretical section is implemented experimentally by acquiring the output of the light source with a 1:99 coupler and getting rid of it from the AAM-influenced interferometric signals. The newly generated LFs which possess perfect elliptic shapes, along with the low MSEs in the ellipses fitting, demonstrate the proposed method performs very well on suppressing the influence of the AAM. Additionally, the asymmetric parameters are calibrated and monitor using such an ameliorated calibration method for 10 min, which remain nearly unchanged, proving that the proposed method is robust and reliable practically. Last but not least, it is mentioned that the method also plays important roles in eliminating the effect of other intensity noises originated from the laser on the calibration, such as the relaxation oscillation-induced noise and the power-line interference as well.
